# A systematic review and meta-analysis of laparoscopic partial nephrectomy versus laparoscopic focal therapy for small renal masses

**DOI:** 10.1097/MD.0000000000044048

**Published:** 2025-08-15

**Authors:** Wu Dan, Lu Ya, Liu Yang, Lin Dong

**Affiliations:** a Department of Urology, Pengzhou People’s Hospital, Chengdu, Sichuan Province, China; b Department of Nephrology, Pengzhou People’s Hospital, Chengdu, Sichuan Province, China.

**Keywords:** laparoscopic focal therapy, laparoscopic partial nephrectomy, meta-analysis, small renal masses

## Abstract

**Background:**

Laparoscopic partial nephrectomy (LPN) and laparoscopic focal therapy (LFT) have emerged as minimally invasive alternatives for managing small renal masses. Despite their increasing clinical adoption, comparative evidence regarding their clinical profiles remains limited. This systematic review and meta-analysis comprehensively evaluates perioperative outcomes, renal function preservation, and oncological efficacy between these 2 interventions for small renal mass treatment.

**Methods:**

A systematic literature search was conducted across Embase, Cochrane Library, and PubMed to identify comparative studies evaluating LPN versus LFT including radiofrequency ablation, microwave ablation, cryoablation, irreversible electroporation, and stereotactic body radiation therapy. Statistical synthesis was performed using random-effects models to calculate pooled mean differences (MD) and odds ratios with corresponding 95% confidence intervals (CIs) through Review Manager, version 5.2 (The Cochrane Collaboration, Oxford, United Kingdom) Stata v.12.0 (Stata Corp LLC, College Station, TX).

**Results:**

The meta-analysis incorporated 10 studies encompassing 1247 participants. Compared with LPN, LFT demonstrated significantly shorter operating time (MD ‐51.76, 95% CI ‐88.22 to ‐15.30; *I*^2^ = 97%, *P* = .005) and reduced estimated blood loss (MD ‐107.92, 95% CI ‐139.35 to ‐76.48; *I*^2^ = 15%, *P* < .00001). LFT interventions also showed advantages in postoperative recovery, with shorter length of stay (MD ‐1.61; CI ‐2.44 to ‐0.78; *I*^2^ = 91%, *P* = .0001) and better preservation of renal function as measured by estimated glomerular filtration rate (MD ‐8.21; CI ‐10.77 to ‐.64; *I*^2^ = 0%, *P* < .00001). However, LFTs were associated with a 3.42-fold increased risk of local tumor recurrence (odds ratios 3.42; CI 1.25–9.33; *I*^2^ = 0%, *P* = .02). No significant between-group differences were observed in overall complication rates (*P* > .05).

**Conclusion:**

LPN demonstrates superior oncological control with reduced local recurrence risks relative to LFT. Conversely, LFT exhibit significant advantages in nephron preservation, minimized intraoperative hemorrhage, and accelerated length of stay. In the contemporary minimally invasive treatment paradigm, clinical decision-making requires careful consideration of tumor biology, renal functional reserve, and individualized risk-benefit profiles to optimize therapeutic strategies.

## 1. Introduction

The diagnostic paradigm shift driven by advanced cross-sectional imaging modalities (CT/MRI) and intensified health surveillance programs has resulted in a 48.7% increase in incidental detection rates of subclinical small renal masses (SRMs) ≤ 4 cm, as evidenced by SEER database analyses (2015–2020).^[1]^ Advancements in nephron-sparing techniques coupled with improved understanding of SRM tumorigenesis have driven paradigm shifts in surgical management. Contemporary analyses of the National Cancer Database (2015–2020) reveal laparoscopic partial nephrectomy (LPN) now constitutes 68.3% of SRM interventions, surpassing radical approaches in T1a tumors (≤4 cm) per European Association of Urology guidelines.^[2]^ While LPN demonstrates technical proficiency demands (R.E.N.A.L nephrometry score ≥9 cases requiring 23% longer operative time) and inherent procedural morbidity (15.8% Clavien-Dindo ≥ II complications in T1b tumors), this has catalyzed technological evolution in renal preservation strategies. Contemporary urological research has prioritized the development of image-guided ablation modalities (cryoablation/microwave ablation) demonstrating equivalent oncologic outcomes with 36% reduced hospitalization duration, as endorsed by the 2023 American Urological Association guidelines for select cT1a lesions.^[3]^

Among minimally invasive nephron-sparing techniques, localized focal therapy (LFT), including radiofrequency ablation, microwave ablation, cryoablation, and nonthermal irreversible electroporation, has demonstrated promising outcomes.^[4]^ Unlike procedures requiring renal parenchymal incision and vascular clamping, LFT better preserves nephrons and mitigates renal ischemia–reperfusion injury. Nevertheless, concerns persist regarding its safety and efficacy. This meta-analysis evaluates existing literature to compare the clinical efficacy and safety profiles of LPN and LFT, aiming to provide evidence-based guidance for clinical practice.

## 2. Methods

### 2.1. Protocol and guidance

This systematic review and meta-analysis adhered to preferred reporting items for systematic reviews and meta-analysis guidelines,^[5]^ with a prospectively registered protocol on PROSPERO (CRD420250285625).

### 2.2. Search strategy

A comprehensive multi-database search strategy was implemented across PubMed, EMBASE, and Cochrane Central Register of Controlled Trials (CENTRAL) through March 1, 2025, utilizing a structured PICO framework. The search terms (taking Pubmed as an example) included (laparoscopic partial nephrectomy OR LPN OR Minimally invasive) AND (“Renal Neoplasm” [MeSH] OR renal masses) AND (“laparoscopic radiofrequency ablation” [MeSH] OR “laparoscopic cryoablation” [MeSH] OR laparoscopic microwave ablation OR laparoscopic radiofrequency ablation OR laparoscopic irreversible electroporation OR LCRA OR laparoscopic microwave ablation OR LIEP OR ‘ Laparoscopic Stereotactic body radiation therapy’ [MeSH] OR laparoscopic stereotactic body radiation therapy). No language or temporal filters were applied during the initial search phase. Protocol registration was completed via PROSPERO (CRD420250285625), with full methodological exemption from institutional review board oversight per NIH guidelines for secondary data synthesis studies involving de-identified aggregate data.

### 2.3. Inclusion and exclusion criteria

Studies were selected based on the following criteria: provision of comparative data on SRM treatment via LPN versus LFT (laparoscopic radiofrequency ablation, laparoscopic cryoablation, laparoscopic microwave ablation, laparoscopic irreversible electroporation, laparoscopic stereotactic body radiation therapy), and reporting of at least 1 outcome measure among perioperative metrics, renal function, or oncologic outcomes. Noncompliant studies were excluded.

### 2.4. Data extraction and outcome measures

Two investigators (LY and WD) independently screened the retrieved literature against the predetermined inclusion/exclusion criteria. Discrepancies were resolved through discussion with a third researcher (LD). The following data were extracted: first author, publication, country, study type, group, age (if reported), follow-up duration, female proportion, end points, study interval, and renal nephrectomy score (summarized in Table 1).

**Table 1:** The main characteristics of included studies.

Author	Year	End points	Publication	Country	Study design	Study interval	Group	Cases	Age	Male proportion (%)	BMI (body mass index) (kg/m2)	Comorbidities ASA (%)	Tumour size (cm)	Pathology (Ma/Be/Un)	R.E.N.A.L nephrometry score	Follow-up (mo)	Confounders adjustment	NOS score (max: 9)
Bensalah	2007	Survival, recurrence, complications	BJU International	USA	R	2000–2006	LPN	50	56.5 ± 11.7	62	31.1 ± 8.0	≥3 (53%)	2.6 ± 0.9	N/A	N/A	25	No	8
							LRFA	38	62 ± 17.5	58	29.6 ± 4.8	≥3 (26%)	2.3 ± 0.7			15		
Bird	2009	Survival, recurrence, complications, renal function	Journal of Endourology	USA	R	2002–2007	LPN	33	57.8 (27–77)	55	28.45	2.2	3.1	20/13/0	N/A	27 (6–58)	No	7
							LRFA	36	75.2 (56–86)	61	30.08	2.8	2.8	26/13/0		12 (6–23)		
Desai	2005	Survival, recurrence, complications	Urology	USA	P	1999–2003	LPN	153	60.59 ± 13.19	58	29.06 ± 6.42	≥3 (46)	2.25 ± 0.67	N/A	N/A	5.8 (1–36)	No	6
							LCA	89	65.55 ± 12.69	69	27.43 ± 5.59	≥3 (75)	2.05 ± 0.56			24.6 (1–60)		
Haber	2012	Survival, recurrence, complications, renal function	BJU international	USA	R	1998–2008	LPN	48	60.6 ± 13.7	52.1	30.1 ± 6.2	2.7 ± 0.5	3.2 ± 1.33	31/17/0	N/A	42.7 ± 30.8	No	8
							LCA	30	60.9 ± 11.4	73.3	31.5 ± 5.8	2.7 ± 0.8	2.6 ± 1.08	25/5/0		60.2 ± 46.3		
Haramis	2012	Survival, recurrence, complications	Journal of Laparoendoscopic and Advanced Surgical Techniques	USA	R	2005–2008	LPN	92	58.8 (37–85)	60.8	N/A	N/A	1.9 (0.3–4.5)	N/A	N/A	21.8 (1–48)	No	6
							LCA	75	69.2 (19–84)	62.7		1.9 (1–3)	2.0 (0.4–7.5)			14 (1–34)		
Ji	2016	Recurrence, complications	Urologia Internationalis	China	R	2006–2015	LPN	74	57.3 (25–76)	55.4	N/A	1.7 (1–3)	2.9 (1.4–3.8)	103/2/0	N/A	2.2 (1.7–3.3)	No	6
							LRFA	105	64.2 (42–81)	62.9		2.3 (1–3)	2.2 (1.7–3.3)	71/3/0		78 (60–106)		
Kiriluk	2011	Complications, renal function	Journal of Endourology	USA	P	2002–2008	LPN	51	66.0 (23–83)	51	29.1 (18.2–24)	N/A	2.27 (0.80–5.10)	N/A	N/A	18.3 (13.0–26.8)	No	7
							LAT	51	65.7 (27–75)	51	30.0 (12.1–56.9)		2.35 (0.99–4.90)			27.9 (0.4–40.0)		
Lian	2010	Recurrence, complications, renal function	Chinese Journal of Surgery	China	R	2005–2009	LPN	29	61 (55–68)	66	N/A	N/A	2.8 (2.0–4.5)	N/A	N/A	27 (3–36)	No	6
							LCA	18	63 (41–73)	78			2.9 (1.5–5.0)			16 (6–21)		
Link	2006	Recurrence	Journal of Endourology	USA	R	2004–2005	LPN	217	N/A	N/A	N/A	N/A	2.6 ± 1.3	N/A	N/A	N/A	No	7
							LCA	28					2.4 ± 0.9					
O’Malley	2007	Recurrence, complications	BJU International	USA	R	2003–2005	LPN	15	75.7 ± 4.6	79	27.1 ± 3.9	≥3 (53)	2.5 ± 1.0	N/A	N/A	9.83 ± 8.8	Yes: matching	8
							LCA	15	76.1 ± 4.5	57	29.1 ± 6.8	≥3 (62)	2.7 ± 1.3			11.9 ± 7.2		

Matching: 1 = age, 2 = BMI, 3 = ASA, 4 = Charlson, 5 = gender, 6 = pathological stage, 7 = urinary diversion type.

CRA = cryoablation, LPN = laparoscopic partial nephrectomy, MWA = microwave ablation, NA = data not available, NOS score = Newcastle–Ottawa Scale score, P = prospective, R = retrospective, RARC = robot-assisted partial nephrectomy, RCT = randomized controlled trial, RFA = radiofrequency ablation.

### 2.5. Statistical analysis

Statistical analyses were conducted using Review Manager (v5.2; Cochrane Collaboration, Oxford, UK) and Stata (v12.0; StataCorp LLC, TX). Heterogeneity thresholds were predefined as *I*^2^ > 50% with *P* < .1, for random-effects model application, and *I*^2^ ≤ 50% with *P* ≥ .1 for fixed-effects models. Pooled risk ratios with 95% confidence intervals (CIs) were computed, and study-specific outcomes were visualized via forest plots. Study quality was assessed with the Newcastle–Ottawa Scale, while publication bias was evaluated using Begg and Egger tests. Statistical significance was defined as *P* < .05.

## 3. Results

### 3.1. Eligible studies and study characteristics

**Figure 1.**
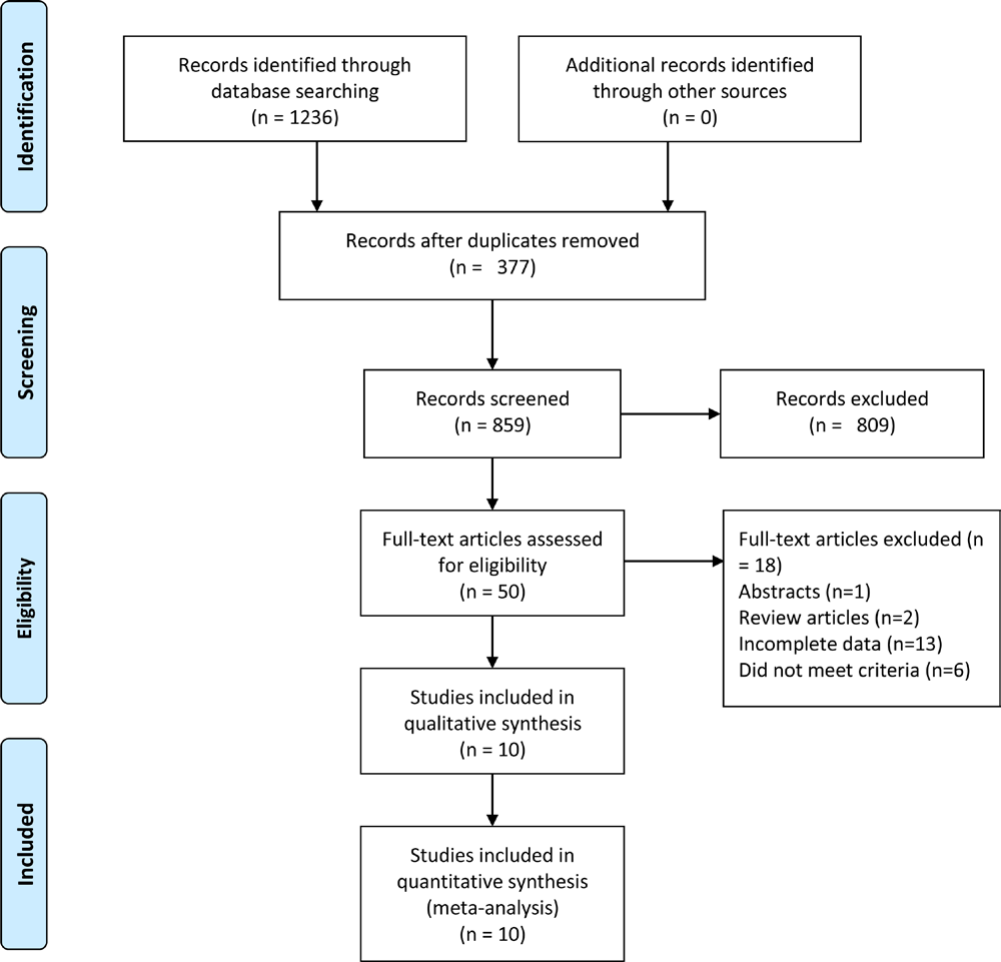
Flowchart for records selection process of the meta-analysis (according to PRISMA template: Moher et al^[16]^).

The initial database search identified 1236 publications. After removing 377 duplicate records, 709 articles were excluded through title/abstract screening. Full-text assessment of the remaining 50 articles led to the final inclusion of 10 studies^[6–15]^ (N = 1274; LPN: n = 762, LFT: n = 512), with detailed characteristics presented in Table 1. The selection process is summarized in Figure 1.

### 3.2. Perioperative outcomes

**Figure 2.**
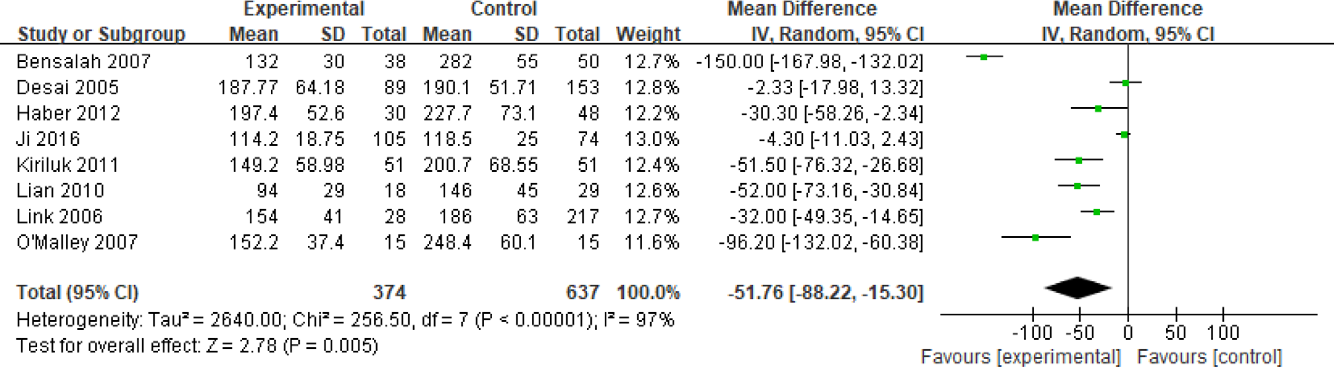
Forest plots of perioperative outcomes: operating time.

Eight studies^[6–10,12,13,15]^ reported operative period (OP) outcomes. Compared with LPN, the LFT cohort demonstrated significantly reduced OP (mean difference [MD] ‐25.67, 95% CI ‐30.77 to ‐20.58; *I*^2^ = 97%, *P* < .00001). Due to persistent heterogeneity (*I*^2^ = 97%) despite sensitivity and subgroup analyses, and considering the nature of OP outcomes, a random-effects model was applied (MD ‐51.76, 95% CI ‐88.22 to ‐15.30; *P* = .005), with results illustrated in Figure 2.

**Figure 3.**
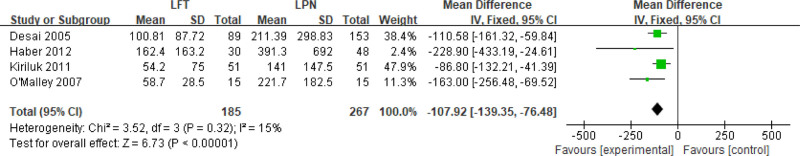
Forest plots of perioperative outcomes: estimated blood loss.

Seven studies^[6,8–10,12,13]^ evaluated estimated blood loss. The LFT group demonstrated significantly reduced estimated blood loss versus LPN (MD ‐107.25, 95% CI ‐121.25 to ‐93.47; *I*^2^ = 96%, *P* < .00001). Given substantial heterogeneity (*I*^2^ = 96%), sensitivity analysis was performed. Sequentially excluding Lian et al^[8]^ and Ji et al^[10]^ maintained stable pooled estimates (MD ‐107.92, 95% CI ‐139.35 to ‐76.48) while markedly lowering heterogeneity (*I*^2^ = 15%, *P* < .00001), as detailed in Figure 3.

**Figure 4.**
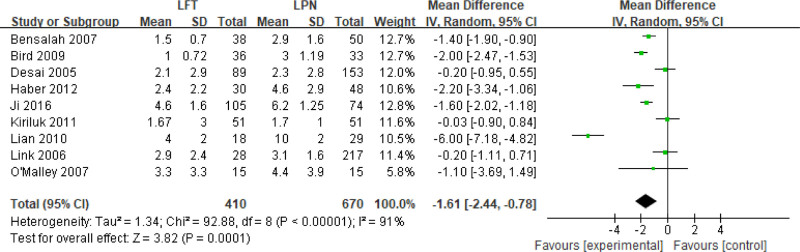
Forest plots of perioperative outcomes: length of stay.

Nine studies^[6–10,12–15]^ reported length of stay (LOS) outcomes. Initial fixed-effects modeling revealed substantial heterogeneity between LPN and LFT groups (*I*^2^ = 91%). As sensitivity and subgroup analyses failed to reduce heterogeneity, and considering the clinical characteristics of LOS outcomes, a random-effects model was applied (MD ‐1.61, 95% CI ‐2.44 to ‐0.78; *P* = .0001), with results visualized in Figure 4.

**Figure 5.**
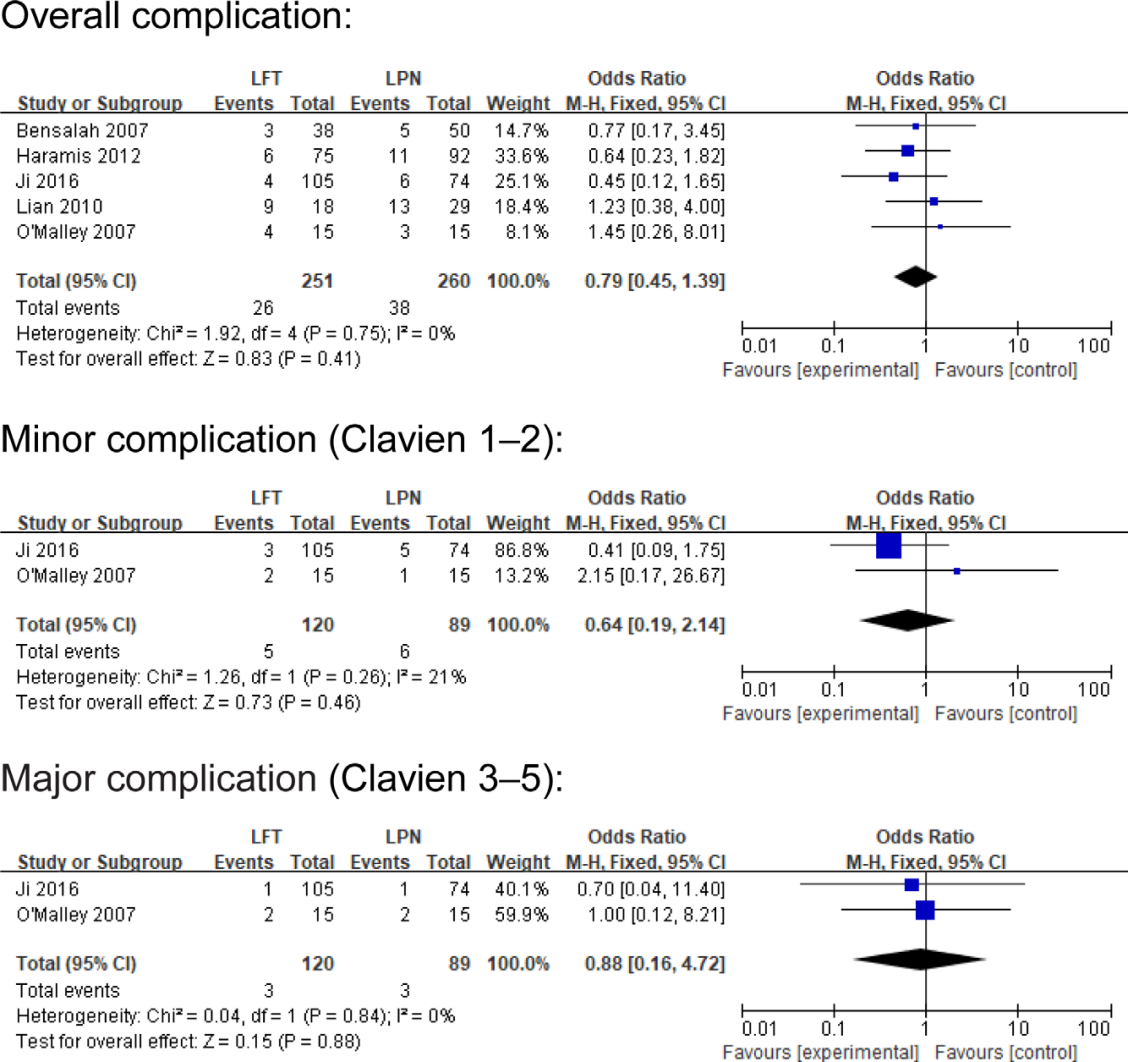
Forest plots of perioperative outcomes: minor complication (Clavien 1–2), major complication (Clavien 3–5), and overall complication.

A fixed-effects model was applied for complication analyses. Minor complication (Clavien 1–2) (odds ratios [OR] 0.64; CI 0.19–2.14; *I*^2^ = 21%, *P* = .46), major complication (Clavien 3–5) (OR 0.88; CI 0.16–4.72; *I*^2^ = 0%, *P* = .88), and overall complication (OR 0.79; CI 0.45–1.39; *I*^2^ = 0%, *P* = .41) were similar between LPN and LFT (Fig. 5).

### 3.3. Renal functional outcomes

**Figure 6.**
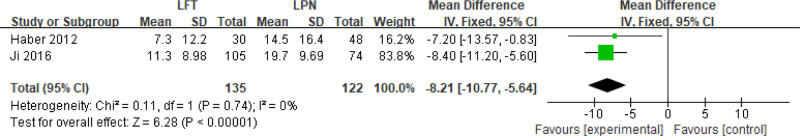
Forest plots of perioperative outcomes: eGFR. eGFR = estimated glomerular filtration rate.

Functional outcomes were assessed through systematic analysis of estimated glomerular filtration rate. Three studies^[10,12,15]^ provided estimated glomerular filtration rate data, revealing significantly lower values in LFT recipients versus LPN (MD ‐8.69, 95% CI ‐11.25 to ‐6.13; *I*^2^ = 96%, *P* < .00001). Given substantial heterogeneity (*I*^2^ = 96%), sensitivity analysis was conducted. Sequential exclusion of Bensalah et al^[15]^ maintained consistent pooled estimates (MD ‐8.21, 95% CI ‐10.77 to ‐5.64) while eliminating heterogeneity (*I*^2^ = 0%, *P* < .00001), as shown in Figure 6.

### 3.4. Oncological outcomes

**Figure 7.**
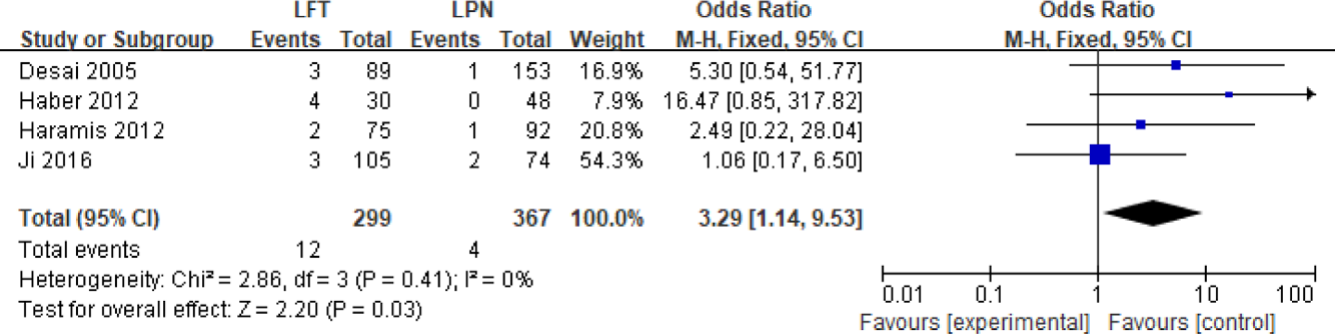
Forest plots of perioperative outcomes: local recurrence rate.

Oncological outcomes were monitored over median/mean follow-up periods of 2.2 to 42.7 months (LPN) and 12 to 78 months (LFT). Local recurrence rates were assessed in 5 studies, demonstrating significantly higher risk with LPN versus LFT (OR 3.29, 95% CI 1.14–9.53; *I*^2^ = 0%, *P* = .03; Fig. 7). Distant metastasis rates were reported in only 1 study, precluding comparative analysis.

## 4. Publication bias

Although publication bias assessment using Egger test was planned for analyses with ≥ 10 studies, this method was precluded as no meta-analysis included sufficient data (all had < 10 studies).

## 5. Discussion

Laparoscopic local therapy involves tumor localization under laparoscopic guidance followed by multimodal ablation techniques. These induce coagulative necrosis of renal tumor cells, with cryoablation zones monitored in real-time via direct vision and ultrasound guidance. Ensuring cryoablation margins extend ≥1 cm beyond tumor boundaries is critical for complete lesion eradication, substantially mitigating residual tumor risk.^[17]^ Moreover, the self-antigens released by frozen necrotic tissue cells can induce autoimmune reactions, thereby playing a role in immune regulation.^[18]^ Although clinical interest in laparoscopic local therapy is growing, persistent debates concerning its procedural safety and efficacy underscore the need for systematic evidence synthesis.

Treatment selection between partial nephrectomy (PN) and focal therapy (FT) necessitates integrated assessment of patient-specific factors, including comorbidities, life expectancy, tumor characteristics (size, multiplicity, and anatomical complexity), renal function staging, and concurrent medical conditions.^[19,20]^ Literature indicates typical tumor diameters of 1.9 to 2.7 cm for ablative procedures, with select series extending to 7 cm.^[21,22]^ FT cohorts demonstrate significantly smaller lesions, less anatomically challenging locations, higher incidence of multifocal tumors, and predominantly endophytic growth patterns compared to PN.^[23,24]^ Among SRM patients receiving ablation, 91% to 97% exhibited low-to-intermediate procedural complexity based on R.E.N.A.L nephrometry scoring.^[25,26]^ Notably, no included studies formally evaluated surgical complexity using standardized nephrometry systems. Clinical decision-making regarding PN versus FT should incorporate Charlson Comorbidity Index and tumor complexity scores alongside conventional parameters (e.g., age, tumor size) to optimize therapeutic benefit.^[27]^

This meta-analysis reveals that LFT demonstrates superiority in preserving renal function, minimizing blood loss, and reducing hospital stay duration. Conversely, LPN shows a significant edge in preventing tumor recurrence. When evaluating renal function changes using serum creatinine and glomerular filtration rate as primary indicators, LFT exhibits minimal impact on the affected kidney’s function and outperforms LPN in renal preservation. These benefits likely stem from LFT’s avoidance of ischemia–reperfusion injury, precise targeting, elimination of renal parenchymal transection, and shorter operative times. Furthermore, the higher intraoperative blood loss associated with LPN may be attributable to tumor resection and the subsequent dissection and suturing of vessels within the wound bed.

This study reported median follow-up durations of 5.8 to 82 months for LPN and 12 to 78 months for LFT. Meta-analysis indicated a higher risk of tumor progression in the LFT group, characterized by increased rates of local recurrence and distant metastasis relative to LPN. Contrastingly, Turna et al^[28]^ observed comparable 2-year overall survival and cancer-specific survival between the groups. Aron et al’s findings further support LFT, reporting 5-year and 10-year overall survival, cancer-specific survival, and disease-free survival rates of 84%/92%/81% and 51%/83%/78%, respectively, in 80 LFT-treated SRM cases, demonstrating effective long-term tumor control.^[29]^ Collectively, these results suggest comparable short-to-medium term efficacy for LFT versus LPN in SRM management. While standalone LFT studies report acceptable long-term outcomes, robust comparative trials with extended LPN follow-up are lacking. Our meta-analysis identified poorer local control with LFT, potentially attributable to the precise tumor delineation and resection under direct vision afforded by LPN surgery, whereas LFT relies on cryoablation ice margins extending 1 cm beyond the tumor for necrosis. Additionally, clinical decision-making introduces significant selection bias, as older patients, those with solitary kidneys, renal impairment, or significant comorbidities are often preferentially directed towards LFT.

This meta-analysis is subject to several limitations. Primarily, the included studies consist exclusively of observational designs, lacking randomized controlled trials. Consequently, the findings may be susceptible to inherent biases and confounding factors associated with such studies. Furthermore, significant heterogeneity was observed for operative time and LOS. Despite sensitivity and subgroup analyses, the sources of heterogeneity remained unresolved, necessitating the use of a random-effects model. Finally, the scarcity of large-scale, long-term comparative studies impedes a comprehensive assessment of long-term oncologic outcomes.

## 6. Conclusion

LPN demonstrates superior local tumor control compared to LFT. Conversely, LFT excels in renal function preservation, hemorrhage reduction, and shortening hospitalization. Within the minimally invasive paradigm, treatment selection requires holistic evaluation of these trade-offs to guide personalized decisions.

## Author contributions

**Conceptualization:** Wu Dan.

**Data curation:** Wu Dan.

**Formal analysis:** Wu Dan.

**Funding acquisition:** Wu Dan.

**Investigation:** Wu Dan.

**Methodology:** Lu Ya.

**Project administration:** Lu Ya, Lin Dong.

**Resources:** Lu Ya, Liu Yang.

**Software:** Liu Yang.

**Supervision:** Liu Yang.

**Validation:** Liu Yang.

**Writing – original draft:** Lin Dong.

**Writing – review & editing:** Lin Dong.
